# Plant-Based Flavones of Therapeutic Interest Loaded into Polymeric Nanoparticles

**DOI:** 10.3390/pharmaceutics18060676

**Published:** 2026-05-29

**Authors:** Cecilia Espíndola, Ira Wirth, Victoria Isabel Martín, Eva Bernal, José Antonio Lebrón, María Luisa Moyá, Rafael R. de la Haba, Cristina Sánchez-Porro, Antonio Ventosa, Carmen M. Granados-Carrera, Sara Molina, Alvaro Hidalgo, Manuel López-López, Pilar López-Cornejo, Francisco José Ostos

**Affiliations:** 1 Department of Physical Chemistry, Faculty of Chemistry, University of Seville, C/Profesor García González 1, 41012 Seville, Spain; caced0617@gmail.com (C.E.); ira.wirth@uni-hamburg.de (I.W.); vmartin1@us.es (V.I.M.); evabernal@us.es (E.B.); jlebron@us.es (J.A.L.); moya@us.es (M.L.M.); saramolinavz@gmail.com (S.M.); alvhidyer@alum.us.es (A.H.); fostos@us.es (F.J.O.); 2 Department of Microbiology and Parasitology, Faculty of Pharmacy, University of Seville, C/Profesor García González 2, 41012 Seville, Spain; rrh@us.es (R.R.d.l.H.); sanpor@us.es (C.S.-P.); ventosa@us.es (A.V.); 3 Department of Chemical Engineering, Faculty of Chemistry, University of Seville, C/Profesor García González 1, 41012 Seville, Spain; cargracar@alum.us.es; 4 Department of Chemical Engineering, Physical Chemistry and Materials Science, Faculty of Experimental Sciences, Campus de El Carmen, Avda. de las Fuerzas Armadas s/n, 21007 Huelva, Spain

**Keywords:** polymeric nanoparticles, PLGA, flavones, drug release, EC_50_, MIC

## Abstract

**Background/Objectives**: Flavonoids are low-molecular-weight polyphenolic compounds that are universally distributed in plants. They are a chemically varied group of secondary metabolites with a broad range of biological activity. The use of flavonoids is known to decrease the risk of many chronic diseases due to their radical scavenging, antioxidant, anti-inflammatory, anticarcinogenic, and antimutagenic properties. Limitations in the use of flavonoids include their low water solubility and poor stability, and therefore their low bioavailability. The encapsulation of flavonoids in different nanocarriers has helped to overcome this limitation. Taking this into account, in this work, the encapsulation of four flavones with several therapeutic applications—7-hydroxyflavone, 7,8-dihydroxyflavone, baicalein, and luteolin—in poly(lactic-co-glycolic) acid (PLGA)-derived polymeric nanoparticles (NPs) has been investigated. **Methods**: A physicochemical characterization of the NPs has been carried out using different techniques, including the evaluation of antioxidant and antimicrobial activities. **Results**: In all cases, the encapsulation efficiency of the four flavones in the prepared NPs was high (>90%), the zeta potential was about −31 mV, and the size was nanometric (~450 nm). The drug release from the nanoparticles was also studied, showing first-order kinetics. Statistical tools were applied to the release rate constants. The antioxidant activity and the in vitro antimicrobial activity of the free and flavone-loaded NPs were investigated, in the case of the latter using Gram-positive and Gram-negative bacteria. Results show that when the flavones are encapsulated, they retain their therapeutic properties. **Conclusions**: In summary, PLGA-based NPs not only prevent flavone degradation but also significantly boost solubility, ultimately optimizing bioavailability. Our results underscore these NPs as a promising platform for efficient flavone delivery.

## 1. Introduction

The use of nanostructures as drug nanovehicles has been of interest for many researchers because, frequently, a substantial increase in drug solubility, and therefore bioavailability, is observed. In addition, improvement in oral absorption and lower drug degradation have also been found, enhancing the drug’s therapeutic effects [1,2]. Nanocarriers can be classified as organic, inorganic, and hybrid [1], and they include liposomes, polymeric micelles, nanoemulsions, polymeric nanoparticles, metallic nanoparticles, carbon nanotubes, quantum dots, etc. The nanocarriers’ utility depends on their physicochemical properties, such as size, shape, loading capacity, zeta potential, or composition.

**Figure 1:**
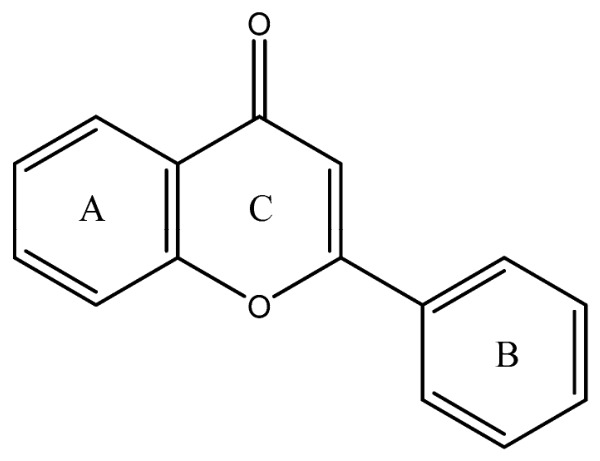
Basic skeleton of flavones.

Our group has been working on an investigation of the encapsulation and release of different drugs in several nanocarriers [3,4,5,6,7,8,9]. Flavones have attracted our interest due to their favorable effects on various diseases such as Alzheimer’s disease, atherosclerosis, cancer, etc. [10,11,12]. Flavonoids are present in a wide range of nutraceutical, medicinal, cosmetic, and pharmaceutical applications. This is mainly due to their anti-oxidative, anti-inflammatory, anti-carcinogenic, and anti-mutagenic properties, as well as their ability to regulate key cellular enzyme functions [13,14,15,16]. The basic structure of flavones is shown in Figure 1. There is a benzo c-pyrone skeleton, which consists of two aromatic rings (A and C), linked through a linear three-carbon chain, forming a closed pyran ring (B), where a keto group is present. Modifications of the flavones’ chemical backbone, including hydroxylation, O-/C-glycosylation, O-methylation, and acylation [13], result in the high degree of chemical diversity of flavones.

**Figure 2:**
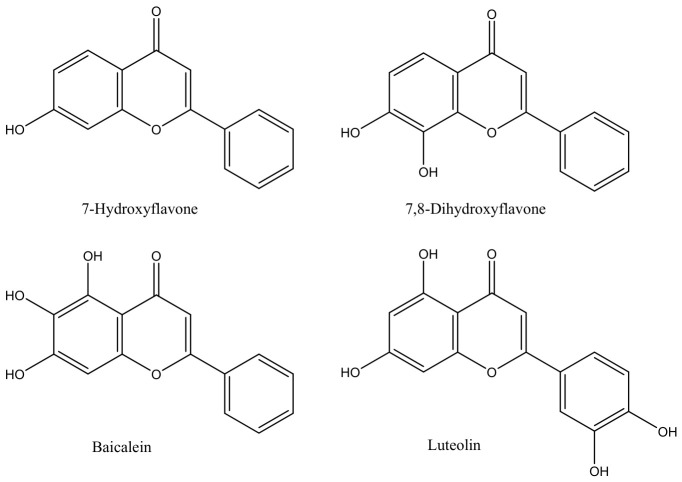
Structure of the flavones studied in this work.

Flavones present low water solubility, and in many cases low stability, and, therefore, low bioavailability. The use of nanoparticle-based delivery systems is a strategy to overcome these limitations. Poly(lactic-co-glycolic) acid (PLGA) is an extensively used polymer due to its biocompatibility and biodegradability. In fact, its clinical use is approved by the FDA and EMA agencies. The use of polyethylene glycol (PEG) to modify the surface of PLGA nanoparticles (NPs) or the utilization of the polymer (polyethylene glycol) methyl ether-block-poly(lactide-co-glycolide) (PLGA-PEG) to prepare the NPs could improve the circulation time and affect the encapsulation efficiency and drug release. In this work, four flavones have been chosen to study their encapsulation in PLGA-derived NPs (see Figure 2). All of them have interesting antioxidant, antiviral, anti-inflammatory, and anticancer activities [17,18,19,20,21,22,23,24,25,26]. In addition, there is an increase in the number of hydroxyl groups present in the flavones from one to four. This will provide an opportunity to investigate how polarity, as well as the hydrogen bond capacity, can influence their interaction with the polymeric NPs’ matrices. Comparison of the physicochemical characteristics and release profiles of the different PLGA-derived NPs investigated can provide information about their performance as drug nanocarriers. These studies will permit the optimization of nanovehicles for flavonoid therapeutics.

Finally, it is worth noting that this investigation is part of a wider study including the utilization of liposomes and carbon nanotubes as nanovehicles for the same flavones [6,7], with the goal of determining which of them would be more suitable for encapsulating and releasing drugs.

## 2. Materials and Methods

### 2.1. Materials

Poly(lactic-co-glycolic) acid (PLGA) (Resomer^®^ RG 502 H), (polyethylene glycol)methyl ether-block-poly(lactide-co-glycolide), PLGA-PEG, polyvinyl alcohol, PVA (with an average molecular weight within the range 30,000–70,000 Da), polyethylene glycol, PEG (with an average molecular weight of 8000 Da), baicalein, BA, and 7-hydroxyflavone, 7-HF were purchased from Sigma-Aldrich (St. Louis, MO, USA). Luteolin, LU, and 7,8-dihydroxyflavone, 7,8-DHF were from TCI (Tokyo, Japan). The flavones were used as received. The remaining reagents utilized in the preparation of the NPs as well as in the preparation of the buffers were also from Sigma-Aldrich (St. Louis, MO, USA).

Three buffered solutions were prepared. The acid buffer at pH = 2 was 8.06 mM in NaCl and 1.9 mM in HCl. The neutral buffer at pH = 7.4 was 22.7 mM in Tris and 9.8 mM in HCl. The basic buffer solution at pH = 8.2 was 3.7 mM in NaH_2_PO_4_ and 22.7 mM in Na_2_HPO_4_. The pH was adjusted using a Basic 20+ pH meter from Crison Instruments (Barcelona, Spain). These pH values correspond to those in the stomach, the blood stream and the duodenum, and the colon, respectively. MilliQ deionized water (resistivity > 18 MΩ) was used.

Four bacteria were used in antimicrobial susceptibility studies. Gram-negative bacteria included *Escherichia coli* CECT 101 and *Pseudomonas aeruginosa* ATCC 27853. Gram-positive bacteria included *Bacillus subtilis* CCM 2216 and *Staphylococcus aureus* ATCC 27697. These reference strains were obtained from the CECT (Spanish Culture Collection of Type Cultures, University of Valencia, Valencia, Spain), the ATCC (American Type Culture Collection, Manassas, VA, USA), and the CCM (Czech Collection of Microorganisms, Masaryk University, Brno, Czech Republic).

### 2.2. Preparation of Polymeric Nanoparticles

#### 2.2.1. PLGA Nanoparticles

An emulsion/evaporation method was used for 7-HF, 7,8-DHF, and BA [27], where the organic and aqueous phases were immiscible. The polymer (50 mg) and the flavone (different amounts; see Tables 1–4) were dissolved in ethyl acetate (the organic phase). PVA 1.5% *w*/*v* was the aqueous phase. Then, 5 mL of the organic phase, containing the polymer and the flavone, was added over 20 mL of the aqueous phase under magnetic stirring (600 rpm). The resultant dispersion was kept overnight under magnetic stirring until total evaporation of the organic phase. Subsequently, to assure a total evaporation of the organic phase and avoid leaving any traces of solvent contamination in the samples, the remaining dispersion was introduced in an Eppendorf Concentrator plus for 40 min, under vacuum, at room temperature. Afterwards, the polymeric NPs were retrieved by centrifugation over 30 min at 13,500 rpm. The nanoparticles were washed twice with water, and the volume of the supernatants, where the nonencapsulated flavones remained, was measured. These solutions were frozen and kept in the dark. The NPs were dried using an Eppendorf Concentrator plus under vacuum for 2–3 h, at room temperature. The NPs were kept in a freezer in the dark.

Luteolin is not soluble in ethyl acetate and so tetrahydrofuran (THF) was used as organic phase. Since water and THF are totally miscible, the preparation method of the LU-loaded NPs was nanoprecipitation [27]. The procedure was similar to the emulsion/evaporation method described above, that is, 5 mL of the THF organic phase containing 50 mg of the polymer and LU were added to 20 mL of PVA 1.5%, under magnetic stirring (600 rpm). The rest of the preparation, including the evaporation of the organic phase, the two washings, and the subsequent drying of the NPs, was the same as that described for 7-HF, 7,8-DHF, and BA.

#### 2.2.2. PLGA-PEG Nanoparticles

In this case, the polymer, PLGA-PEG, is less soluble in ethyl acetate and THF than PLGA. For this reason, to increase its solubility in the organic phase, 10 µL of deionized water was added to this phase. The rest of the emulsion/evaporation and nanoprecipitation procedures followed were those described in Section 2.2.1.

#### 2.2.3. PLGA+PEG Nanoparticles

The emulsion/evaporation and nanoprecipitation procedures followed were similar to those described in Section 2.2.2 with only one difference: 3.3 mg of the polymer PEG was dissolved in the PVA aqueous phase.

The stability of all prepared nanoparticles was evaluated at 4 °C by determining both their size and zeta potential values. No changes were observed over a period of 2–3 months.

### 2.3. Stability of the Solutions

The stability of the flavones was investigated in water as well as in the three buffered solutions used. To do so, UV-visible and emission fluorescence spectra, depending on the antioxidant nature, were registered as a function of time. The results showed that, if the solutions remained in the dark, they were stable for at least more than two hours. The stability depends on the pH, following the trend: basic media < neutral media ≈ acid media. All solutions were freshly prepared before use and kept in the dark.

### 2.4. Fluorescence Measurements

Fluorescence emission spectra of 7-HF were registered in a Hitachi F-2500 spectrofluorimeter (Hitachi, Tokyo, Japan), connected to a flow Lauda thermostat. A 10 mm path length quartz cell was used. The excitation wavelength was 350 nm, and the spectra were recorded from 400 to 650 nm. The excitation and emission slits were 2.5 nm and 5 nm, respectively.

With the goal of quantitatively determining the 7-HF concentration encapsulated (or released) into the NPs, calibration curves in water as well as in the three buffers (pH values 2.0, 7.4, and 8.2) were obtained. To do so, a concentrated 7-HF ethanol solution was prepared. This solution was kept in the dark and at 4 °C to avoid the flavone degradation. The standard solutions were prepared by adding to a 10 mL flask an adequate aliquot of the ethanolic solution; subsequently, the ethanol was evaporated with an air flow. Then, the flask was leveled with deionized water or with buffer and introduced in a sonicator for 20 min in the dark. The concentration of the standard solutions was within the range 2.52 × 10^−7^ M to 7.20 × 10^−5^ M.

### 2.5. UV-Visible Spectroscopic Measurements

The spectra of the different solutions were registered in a Hitachi U-3900 connected to a water flow Lauda cryostat. A 10 mm quartz cell was used to measure the absorbance.

The wavelengths used to measure the absorbance depended on the species as well as on the buffer. The absorbance of LU was measured at 350 nm in acid and neutral buffers, and at 400 nm in a basic buffer. The standard solution concentrations used to do the calibration curves were within the range 2.00 × 10^−6^–6.00 × 10^−5^ M in the three buffers. For BA, the absorbance was measured at 322 nm in acid buffer and at 358 nm in neutral and basic buffer. The standard solution concentrations used for doing the calibration curves in the three buffers were within the range 1.00 × 10^−5^–7.50 × 10^−5^ M. Finally, for 7,8-DHF, absorbance was measured at 265 nm in acid buffer, and at 300 nm in neutral and basic buffer. The calibration curves were achieved using standard solution concentrations within the range 3.00 × 10^−5^–10.0 × 10^−5^ M, in the three buffers.

A calibration curve was also measured for LU, BA, and 7,8-DHF in water, for determining the flavone concentrations in the supernatants and washings after the NPs’ preparation. The absorbance was measured at the same wavelength as that used in neutral buffer. The standard solution concentration ranges were the same as those mentioned above.

### 2.6. Size and Zeta Potential

The size distribution and the zeta potential (ζ) of the NPs prepared were examined using a Malvern Zetasizer Nano ZSP instrument (Malvern Panalytical, Great Malvern, UK). A scattering angle of 90° was used in the size distribution studies. At least six repetitions were made for each sample, and the average value was considered.

### 2.7. Transmission Electron Microscopy

A Talos X200 microscope (Thermo Fisher Scientific, Waltham, MA, USA) was used to carry out the transmission electron microscopy, TEM, experiments. The samples were prepared as follows. A drop of an aqueous dispersion of the NPs (~1 mg/mL) was added to a copper grid coated with a carbon film and air dried at room temperature.

### 2.8. Fourier Transform Infrared Spectroscopy (FTIR)

FTIR spectra were run for the determination of the molecular composition and intermolecular interactions between polymer and flavone liposomes, by using a Hyperion 100 spectrometer (Bruker, Billerica, MA, USA) equipped with an ATR diamond sensor. Spectra were obtained between 4000 and 400 cm^−1^ with an average of 200 scans and 4 cm^−1^ resolution.

### 2.9. Encapsulation Efficiency, % EE

Equation (1) was used to calculate the encapsulation efficiency (% EE):

(1) $$\%\;\mathrm{EE}=\frac{mg\;of\;encapsulated\;flavone}{total\;mg\;flavone}\times 100$$

where total mg flavone is the amount of flavone initially present in the preparation of the NPs. Once the loaded NPs were removed by centrifugation, the amount of flavone encapsulated was estimated by subtracting the amount of drug left in the supernatant and washings from the initial amount of flavone present in the synthesis. The amount of 7-HF was determined recording the fluorescence emission intensity spectra, while 7,8-DHF, BA, and LU were estimated using the corresponding absorbance spectra.

The yield of the different NPs’ syntheses was calculated using Equation (2):

(2) $$\%\;R=\frac{mg\;NPs}{mg\;polymers\;+\;mg\;flavone}\times 100$$

where mg NPs indicates the mg of nanoparticles obtained in each synthesis, and mg of polymers and mg of flavone indicate the initial amounts of polymer (or of polymers in the case of PLGA+PEG) and flavones used in the preparation, respectively.

### 2.10. In Vitro Drug Release

A solution containing the loaded NPs (~1.5 mg/mL) was suspended in the corresponding buffer (pH values 2, 7.4, and 8.2) in a thermostatized glass vial at physiological temperature (37 °C), under dark and continuous magnetic stirring (200 rpm). At determined time intervals, the solution was centrifuged at 13,500 rpm for 30 min to isolate the remaining NPs. Subsequently, a sample of the supernatant was removed and replaced with an equal volume of the buffer. In this way, the in vivo removal of the flavonoids in the systemic circulation is mimicked. The flavone concentration present in the sample was calculated, using either fluorescence emission intensity or absorbance depending on the flavone nature, as was described above. Each experiment was repeated three times, and the average value was considered.

### 2.11. Measurement of the Antioxidant Activity

#### 2.11.1. Antioxidant Activity of Free Flavonoids

The antioxidant activity of the free flavones was estimated using the 2,2-diphenyl-1picrylhydrazyl (DPPH·) radical scavenging method [28]. DPPH· is a nitrogen-centered radical, which presents a UV-visible spectrum with a maximum absorbance of around 515 nm in methanol. The experiments were carried out in the presence of an excess of DPPH·, to ensure that the H donating of the flavones is exhausted. The DPPH· concentration of the methanolic solution was kept constant at 6.30 × 10^−5^ M, while the methanolic flavone solution concentrations were varied within a range dependent on the flavone nature.

The procedure was as follows: 60 µL of each methanolic flavonoid solution was mixed with 157.5 µL of the DPPH· stock solution; subsequently, methanol was added up to a final volume of 3 mL. The mixture was homogenized and was kept in the dark for one hour, at room temperature. This time was long enough for the reaction to be completed. Afterwards, the UV-visible spectrum of the solution was recorded and the absorbance of the DPPH· radical was measured at 515 nm. The radical scavenging activity of the different flavones, expressed as % of inhibition, was estimated by using Equation (3) [28]:

(3) $$\%\;Inhibition=\frac{A_{0}-A}{A_{0}}\times 100$$

where *A*_0_ and *A* are the absorbances at 515 nm measured in the absence and in the presence of the flavones, respectively. From the % *Inhibition* values, the percentage of free DPPH· in the solution can be calculated. Finally, the EC_50_ index was used to evaluate their antioxidant activity. This index is defined as the antioxidant concentration needed to reduce by half the absorbance of the DPPH·. The experiments were repeated three times, and the results are the average of the values obtained. Ascorbic acid was used as a reference antioxidant.

#### 2.11.2. Antioxidant Activity of Flavone-Loaded NPs

Around 10 mg of loaded NPs were weighed, and they were dissolved in pure methanol.

The loaded capacity (% *LC*) is defined as:

(4) $$\%\;LC=\frac{mg\;of\;encapsulated\;flavone}{total\;mg\;of\;NPs}\times 100$$

where the total mg of NPs is the total weight of nanoparticles obtained in the preparation of each batch of NPs. From the % *LC*, and the mg of NPs weighed in each experiment, the total mg of the flavone present in the methanolic solution can be calculated and, therefore, their concentration. Subsequently, different aliquots of this methanolic flavone solution were added to an Eppendorf tube, together with an aliquot of the methanolic DPPH· solution in such a way that the final DPPH· concentration was kept constant at 6.30 × 10^−5^ M. The flavone concentration present in the Eppendorf tube was varied within a range dependent on the flavone nature. The concentration ranges were 10–2000 µM for 7-HF, 1–40 µM for 7,8-DHF, 1–25 µM for BA, and 1–20 µM for LU. Subsequently, the Eppendorf tubes were ultrasonicated for 15 min in the dark and left for 1 h, in the dark, at room temperature. This time was long enough for the reaction between the flavone and the DPPH· radical to be completed. The UV-visible spectrum of the solution was recorded, and the absorbance of the DPPH· radical was measured at 515 nm. The radical scavenging activity of the different flavones, expressed as % of inhibition, was estimated using Equation (3). The EC_50_ index was evaluated as described above. Each experiment was repeated three times.

#### 2.11.3. In Vitro Antimicrobial Activity

The broth microdilution method, performed according to the guidelines of the Clinical and Laboratory Standards Institute (CLSI) [29], was followed to determine the minimal inhibitory concentration (MIC). In brief, stock solutions of free flavones were prepared in DMSO at concentrations 100 times the highest concentration to be tested and then tenfold diluted in cation-adjusted Mueller Hinton II broth (CAMHB). Subsequently, the drug dilutions were further five-fold diluted with CAMHB, reducing the solvent concentration to 2% dimethyl sulfoxide (DMSO) and the compound to the two times strength needed in the test. Concurrently, flavone-loaded NPs, as well as unloaded NPs, were directly suspended in CAMHB at 2× the initial concentration to be used for MIC determinations. To avoid NP aggregation, they were sonicated using a Ultrasonicator UP400S with H3 probe (Hielscher Ultrasonics, Teltow, Germany), at 90% amplitude over two cycles of 30 s, with a 30 s pause between them, and with the vial immersed in an ice bath to prevent temperature rise. No filter sterilization was required for stock solutions since they can be assumed to be sterile. The twice drug/NP concentrations were dispensed into the wells of the first column of a 96-microtiter plate and then twofold serially microdiluted in CAMHB throughout the columns. The bacterial strains were grown on Tryptic Soy Agar (TSA) at 37 °C for 24 h. The colonies were directly suspended in 5 mL of sterile CAMHB, and the turbidity adjusted to 0.08–0.1 at 600 nm wavelength (equivalent to a 0.5 McFarland standard). Afterward, the suspension was diluted 1:150 with CAMHB to obtain a working suspension containing ca. 10^6^ cells/mL, which was further inoculated into each well of the microdilution tray. The final inoculum density achieved was 5 × 10^5^ CFU/mL. Blank unloaded NPs, adjusted to the same concentration as the corresponding drug-loaded NPs, were also tested. Growth controls (inoculation in wells without drugs/NPs) and sterile controls (wells without inoculum) were prepared. Microdilution plates were incubated at 37 °C, and the detection of visible growth was read after 16 to 20 h. MICs were defined as the lowest drug concentration that completely inhibits bacterial growth in the wells as detected by the unaided eye. All experiments were carried out in duplicate across three independent biological replicates (*n* = 6).

### 2.12. Statistical Analysis

For the release results, tests were performed using the GraphPad Prism 8.0.1 software program (San Diego, CA, USA). Statistics were determined with a two-way ANOVA and Tukey’s multiple comparisons test. Each experiment was repeated thrice independently.

## 3. Results and Discussion

### 3.1. Physicochemical Characterization

Table 1, Table 2, Table 3 and Table 4 summarize the encapsulation efficiency (% EE), the synthesis yield (% R), the zeta potential (ζ), the hydrodynamic diameter (d_H_), and the polydispersity index (PDI) obtained for the different blanks (NPs prepared in the absence of flavones) and flavone-loaded NPs studied.

The data listed in Table 1, Table 2, Table 3 and Table 4 show that the synthesis yield % R follows the trend % R(PLGA) ≈ % R(PLGA-PEG) > % R(PLGA+PEG) for 7-HF, 7,8-DHF, and BA, although the differences in % R are not large (within experimental errors). The LU-loaded NPs show somewhat lower % R values for PLGA and PLGA-PEG NPs than for the other three flavones. However, LU % R values for PLGA+PEG NPs are similar to those found for the rest of the flavones investigated. This result could be related to the procedure used in the preparation of loaded LU NPs, which is a nanoprecipitation method and not an emulsion/evaporation method [30].

The encapsulation efficiency is practically higher than 90% for all the flavone-loaded NPs prepared. Taking the experimental errors into account, this result suggests that most of the flavones used in the syntheses were encapsulated into the NPs, independently of the number of hydroxyl groups in their structures. This could be explained considering the hydrophobic character of the flavones investigated, which have a strong affinity for the hydrophobic polymers, and so it would be expected for the flavones to remain within the PLGA-based NPs once the organic solvent is eliminated.

All the blanks and flavone-loaded NPs have a negative zeta potential, close to −30 mV, which is independent of the number of OH^−^ groups present in the flavone. The value for the blank NPs (free of flavone) agrees with literature data [30]. This substantially negative zeta potential is a favorable feature of the NPs since it will avoid nanostructure aggregation.

The average size of all the blanks and flavone-loaded PLGA-based NPs prepared is around 450 nm (Table 1, Table 2, Table 3 and Table 4), within experimental errors. Most of the NPs presented a unimodal size distribution, with a polydispersity index within the range 0.4–0.7. Nonetheless, a bimodal size distribution was observed in some cases, such as 7,8-DHF-loaded PLGA NPs. Figure S1 (Supplementary Materials) shows some representative DLS histograms. This higher degree of size heterogeneity obtained for the 7,8-DHF-loaded PLGA nanoparticles with respect to the unloaded NPs, observed for other flavones, is due to the formation of different 7,8-DHF/PLGA nanostructures in the solution. This heterogeneity disappears in the presence of PEG.

Fourier transform infrared spectroscopy (FTIR) was used to evaluate the interactions among molecules involved in cationic and anionic liposome fabrication incorporating different flavones. Figure S2 shows the FTIR spectra of the different nanoparticles incorporating the flavones 7-HF, 7,8-DHF, BA, and LU into PLGA formulations, as well as the spectra obtained when incorporating those flavones into PLGA and PEG formulations obtained through two different methods. Table S1 displays the main peaks and assignments for the molecular vibrations associated with specific functional groups. In general, all the formulations conserve the main bands associated with polymer-based formulations, varying their intensity between polymer–flavone formulations.

First, there is a band attributed to O-H stretching (region I, 3600–3050 cm^−1^), associated with the hydroxyl groups, which display a low intensity due to the hydrophobic nature of PLGA [31]. However, this intensity increases with the incorporation of flavones into the formulation, as a result of the greater phenolic content, forming H bonds between flavones and the polymeric matrix. Later, there is a band associated with aliphatic C-H stretching (region II, 3075–2750 cm^−1^), which confirms the conservation of the aliphatic structure of the polymer (or polymers) involved in the liposomes modified by the presence of the organic compounds (flavones) with an intermediate behavior. Region III (2750–2400 cm^−1^) displays weak signs associated with vibrational combinations and possible contribution from terminal C-H and -COOH bonds.

After that, the main region of PLGA systems can be observed, with region IV attributed to the ether carbonyl group (C=O) stretching at 1800–1400 cm^−1^, whose intensity diminishes with the incorporation of flavones due to the establishment of intermolecular interactions between that group and the hydroxyl/aromatic groups presented in flavones. The breakage is significant in the case of luteolin (PLGA LU) due to the possibility of forming multiple hydrogen bonds, perturbing the chemical environment as observed in the FTIR peaks and modifying chain mobility [32]. Then, region V (1400–1050 cm^−1^) demonstrates the existence of C-H deformation, COO^−^ stretching, and C-O-C stretching, increasing the complexity of the spectrum due to the oxygen aromatic structure. Finally, regions VI, VII, and VIII refer to aromatic ring deformation and skeletal vibration, which can be particularly significant in the case of flavone formulation due to the conjugated aromatic structure (as observed in the structure without flavones) [33,34,35,36,37].

**Figure 3:**
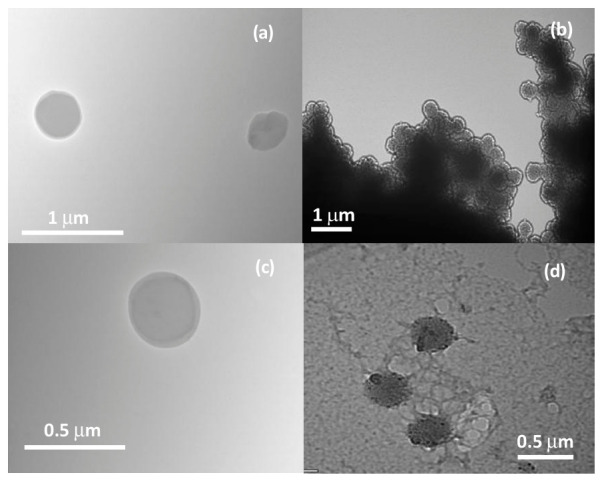
TEM images of various polymeric nanoparticles: (**a**) 7-HF-loaded PLGA NPs (10 mg), size 390 ± 25 nm; (**b**) 7,8-HF-loaded PLGA+PEG NPs (5 mg), size 400 ± 20 nm; (**c**) BA-loaded PLGA-PEG NPs (16 mg), size 390 ± 32 nm; (**d**) LU-loaded PLGA+PEG NPs (2.5 mg), size 400 ± 19 nm.

The morphology of the NPs prepared was investigated by TEM measurements. Figure 3 shows TEM images corresponding to some of the nanoparticles synthesized. In all cases, spherical NPs were observed. The size estimated by TEM (included in the legend of Figure 3) is usually somewhat smaller than that obtained by DLS measurements. This is because size measurements by DLS include the hydration layers. Similar results regarding the spherical shape of PLGA-derived NPs encapsulating several flavonoids have previously been found for other authors, although the NPs’ size depends on the preparation method [38,39,40,41].

The size and the morphology of the NPs prepared with 7-HF, 7,8-DHF, BA, and LU are independent of the number of OH^−^ groups in NPs containing PEG. However, the different size distribution of 7,8-DHF in the absence of PEG indicates a distinct behavior of this flavone, probably due to the position of the -OH groups in its structure. Clearly, PEG polymer stabilizes the nanoparticles.

### 3.2. Flavone Release from the Polymeric Nanoparticles

**Figure 4:**
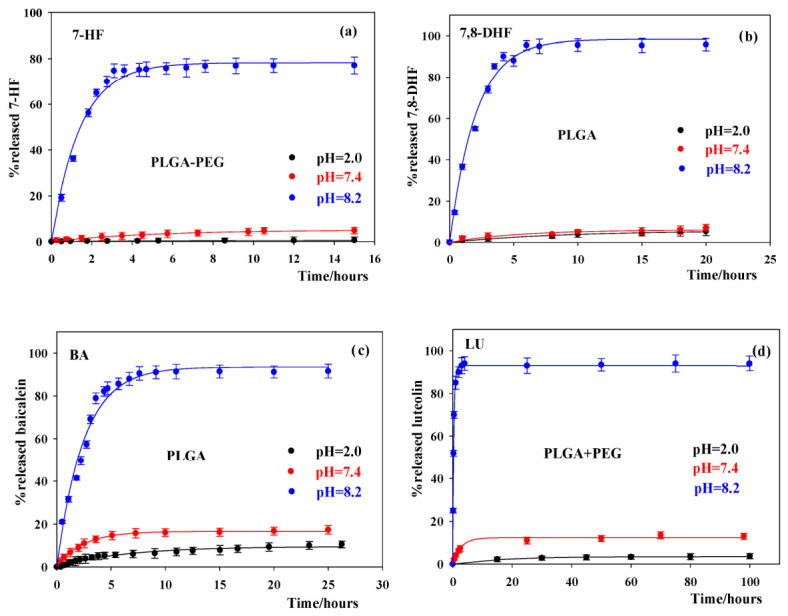
The percentage of cumulative release of the flavones investigated in different PLGA-based NPs in the three buffers used as release media at 37 °C. NP concentration is ~1.5 mg/mL. (**a**) 7-HF, (**b**) 7,8-DHF, (**c**) BA, and (**d**) LU. Solid lines correspond to the fitting of the experimental data using Equation (4).

The percentage of cumulative release in vitro was investigated following the procedure described in Section 2. The release was studied at three different pH values: 2, 7.4, and 8.2, corresponding to those in the stomach, the blood stream and the duodenum, and colon, respectively. Figure 4 shows some examples. In all cases, the percentage of released flavone was found to follow the pH trend: 2 < 7.4 < 8.2. For 7,8-DHF, this percentage is similar in acid and neutral media. Again, the 7,8-DHF shows a different behavior.

Regarding the amount of flavone released at the three pH values investigated, no relationship between the number of hydroxyl groups present in the flavones and the quantity of released drug was observed for 7,8-DHF, BA, or LU. However, for 7-HF, the % of released drug at the three pH values and in all the NPs prepared is lower than those estimated for the other three flavones. This experimental observation could be related to the weaker acid character of 7-HF, as well as its lower water solubility as compared to 7,8-DHF, BA, and LU [42,43,44,45].

The release profiles correspond, in all cases investigated and for the three pH values studied, to first-order kinetic processes. It was checked whether adjusting the data to the sum of more than one first-order paths improved the results, but this was not the case for the different systems investigated. The release rate constants (k_r_) were calculated by using Equation (5):

% Cumulative release drug = *a*·(1 − exp(−k_r_·t)) (5)

where k_r_ is the first-order rate constant corresponding to the drug release, and a is an adjustable parameter. The solid lines in Figure 4 show that the agreement between experimental and theoretical data is good. Comparisons of the release constants as a function of pH, type of NPs, and nature of the flavone are shown in Figures S3–S5 (Supplementary Materials). In general, no clear tendency of k_r_ is observed as a function of the type of NPs. However, the pH has some influence on k_r_. For a given type of flavone-loaded NP, the release rate constant is usually higher in basic media than in acid or neutral media. In relation to the nature of the flavone, no significant differences are observed for 7-HF, 7,8-DHF, BA, or LU in acid and neutral media. Nonetheless, in basic media, the rate of release is much larger for LU than for the other three flavones.

The results in Table 1, Table 2, Table 3 and Table 4 are compared to those previously observed using carbon nanotubes [6,7], as well as phosphatidylcholine/cholesterol liposomes. It was found that carbon nanotubes completely encapsulated the four flavones. However, the encapsulation efficiency of the liposomes was lower. In the case of the carbon nanotubes, the percentage of released flavone was much lower than what was found in this work, and slower too. The release of the flavones from the liposomes was not investigated due to the known low stability of the liposomes with time. Therefore, from the results in this work, it seems that PLGA-based nanoparticles could be considered suitable nanocarriers for the flavones studied.

### 3.3. Antioxidant Activity of the Free and Loaded Flavones

**Figure 5:**
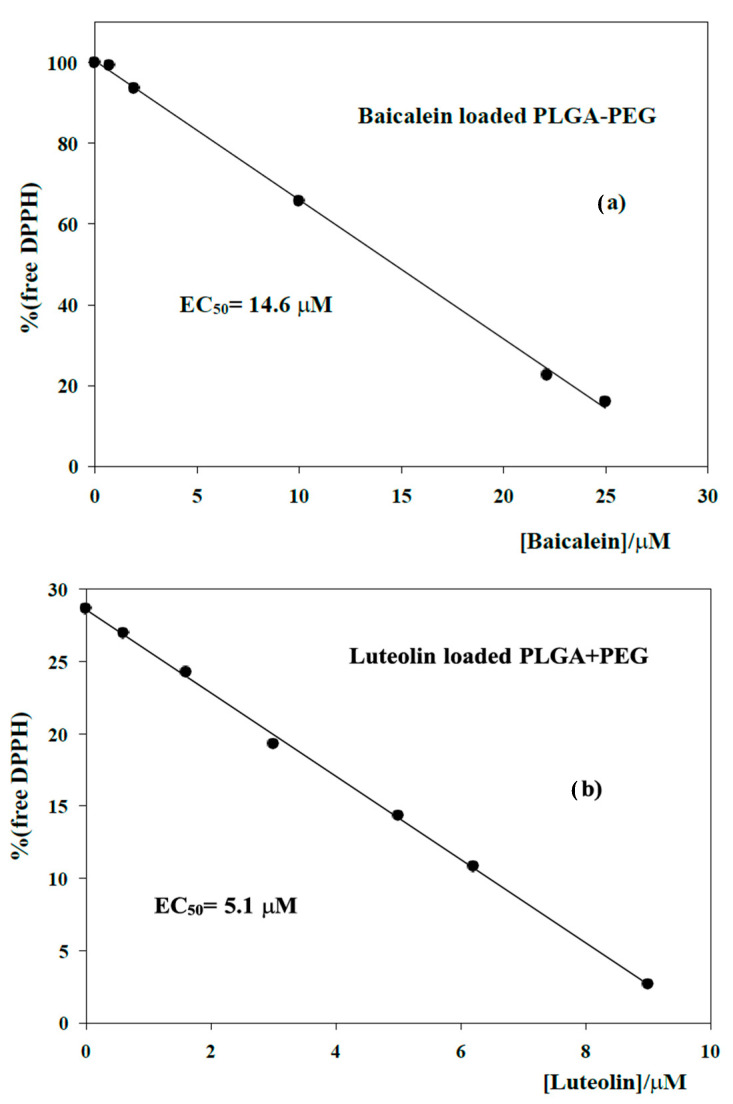
Plots of the % of free DPPH· radical vs. the flavone concentration at room temperature. (**a**) Baicalein loaded into PLGA-PEG NPs; (**b**) Luteolin loaded into PLGA+PEG NPs.

The antioxidant activity of the flavones used in this work, free as well as loaded into the NPs, was estimated by using the DPPH· radical scavenging method [28], as described in Section 2 (see Figure 5). This method has previously been used by other authors to estimate the EC_50_ values of several species loaded into different nanocarriers [46,47,48]. The antioxidant activity was also estimated for ascorbic acid, used as a reference. Table 5 summarizes the EC_50_ values obtained for the systems investigated. The values corresponding to the free flavones agree with the literature data, within experimental errors [48,49,50,51,52]. The observed increase in the antioxidant activity of the free flavones upon augmenting the number of hydroxyl groups present in the molecule agrees with previous results [51,53].

Table 5 shows that 7-HF does not exhibit antioxidant activity either in its free form or loaded into the NPs. Considering the experimental errors, the EC_50_ values of 7,8-DHF and BA increase when they are loaded into the PLGA-based NPs, whereas for LU, the antioxidant activity remains approximately the same once it is loaded into the NPs. The influence of being encapsulated into the NPs on the flavone antioxidant activity depends on the number of OH^−^ groups. For 7,8-DHF and BA, some diminution in antioxidant activity is observed when they are encapsulated, although they are still antioxidant species. Only for LU, which is the one with the highest antioxidant activity, EC_50_ remains constant, within experimental errors. The fact that the loaded flavones retain their antioxidant activity is important since free flavones degrade easily [54,55], and their encapsulation into the PLGA-based NPs avoids this degradation.

### 3.4. Minimum Inhibitory Concentration

The MIC values obtained for the free and loaded flavone NPs in the presence of Gram-positive and Gram-negative microorganisms are summarized in Table 6. Regarding the free flavones, 7-HF does not present antimicrobial activity against either Gram-positive or Gram-negative microorganisms, while 7,8-DHF only shows biocide activity against the Gram-positive *Staphylococcus aureus* ATCC 27697. LU is an efficient biocide against the Gram-positive bacteria, but not against the Gram-negative ones. Its MIC value obtained for *Staphylococcus aureus* agrees with literature data [56]. With regard to BA, it is an efficient antimicrobial agent for both Gram-positive and Gram-negative microorganisms, which means that the biocide activity does not directly depend on the number of OH^−^ groups present in the flavone structure [57]. The MIC value obtained for free BA against *Staphylococcus aureus* ATCC 27697 agrees with literature data [58,59]. Baicalein antimicrobial efficiency was explained by two different mechanisms. Baicalein would form a complex with the cell wall components of the bacteria, consequently inhibiting both further adhesions and microbial growth [51]. Another proposed mechanism was that this flavone was able to downregulate the quorum-sensing system regulators agrA, RNAIII, and sarA, and the gene expression of intercellular adhesin (ica) in bacteria cells [57].

The data in Table 6 show that 7-HF does not present antimicrobial activity when it is encapsulated into PLGA-based NPs. The MIC value for 7,8-DHF-loaded NPs against *Staphylococus aureus* ATTC 27697 is similar to that found for the free flavone. Considering the low antimicrobial activity of 7-HF and 7,8-DHF, the MIC for these two flavones was not determined in PLGA-PEG or PLGA+PEG NPs.

When baicalein is encapsulated in the NPs, the MIC values obtained are similar or somewhat larger than the MIC estimated for the free BA. In the case of luteolin, its encapsulation into the NPs does not practically affect its antimicrobial activity.

Previous studies about the antimicrobial activity of flavonoids encapsulated in polymeric NPs, using a range of different methods, show that, in general, their biocide activity remains approximately similar to that when they are free. The advantage of preparing the loaded NPs is that encapsulation avoids flavonoid degradation [38,60,61,62,63]. Therefore, comparison to results previously found by other authors indicates that the biocide activity of the encapsulated flavonoids in this work follows a similar trend as that found in the literature.

## 4. Conclusions

The results obtained in this work show that the PLGA-based NPs prepared present a high encapsulation efficiency for all the flavones investigated. They have a substantially negative zeta potential, so aggregation of NPs is not expected. They show nanometric size and spherical morphology. A unimodal size distribution was found for most of the flavone-loaded NPs prepared. The formation of different nanostructures of PLGA-based NPs containing 7,8-DHF indicates a distinct interaction between flavone and polymer, probably due to the position of the -OH groups in the flavone. The polymer PEG stabilizes nanoparticles and homogenizes the nanostructure sizes. The size of the nanoparticles obtained (~450 nm) suggests that they are not suitable for parenteral therapeutic applications; however, they may be utilized in oral drug delivery.

The percentage of released flavone from the NPs depends on the pH, following the trend: 2 < 7.4 ≪ 8.2. In basic media, the % of released flavone is close to 100%, with the exception of 7-HF, for which it is a little lower. This observation could be due to the weaker acid character and the lower water solubility of 7-HF as compared to 7,8-DHF, BA, and LU. The release follows a first-order kinetic. No substantial differences in the estimated release rate constants for the four flavones investigated were observed. Therefore, it does not depend on the number of hydroxyl groups.

The antioxidant activity of the free flavones increases as the number of hydroxyl groups augments. The 7-HF does not present antioxidant activity. When the flavones are encapsulated into the PLGA-based NPs, some decrease in the antioxidant activity of 7,8-DHF and BA is observed as compared to the free flavones. As expected, encapsulated 7-HF does not present antioxidant activity. However, LU shows similar EC_50_ values when it is free and when it is loaded into the NPs. Regarding the minimum inhibitory concentrations (MIC), results suggest that the encapsulation can affect the antimicrobial activity of the flavones. For those with two and three hydroxyl groups (7,8-DHF and BA), their biocide activity decreases when they are encapsulated. Nonetheless, when the flavone has four OH^−^ groups (LU), the biocide activity is not affected by the encapsulation into the NPs.

In summary, the high encapsulation efficiency, nanometric size, and unimodal size distribution experimentally observed, together with the fact that flavones’ antioxidant and biocide activities remain operative (although they can change) when they are encapsulated, highlight the suitability of the prepared PLGA NPs as nanocarriers for the encapsulation and delivery of the flavones investigated. This nanoencapsulation avoids degradation, increasing the flavones’ solubility and thus their potential bioavailability.

## Figures and Tables

**Table 1:** Encapsulation efficiency (% EE), synthesis yield (% R), zeta potential (ζ), hydrodynamic diameter (d_H_), and polydispersity index (PDI) values obtained in the preparation of unloaded and 7-HF-loaded polymeric NPs, for different initial amounts of flavone (mg). The experimental values are expressed as the mean ± SD (*n* = 3).

PLGA					
mg 7-HF	% EE	% R	ζ (mV)	d_H_ (nm)	PDI
0	---	90 ± 3	−32.5 ± 1.4	442 ± 10	0.433 ± 0.017
5.4	99 ± 4	96 ± 2	−28.6 ± 1.8	475 ± 34	0.50 ± 0.04
10.0	99 ± 3	84 ± 3	−33.1 ± 1.5	417 ± 29	0.59 ± 0.06
15.3	99 ± 3	93 ± 3	−27.8 ± 0.8	489 ± 42	0.62 ± 0.06
**PLGA-PEG**					
0.0	---	88 ± 2	−28.7 ± 1.8	424 ± 30	0.51 ± 0.04
4.9	99 ± 2	94 ± 2	−27.6 ± 1.2	390 ± 24	0.67 ± 0.07
10.5	99 ± 3	85 ± 4	−29.5 ± 1.5	473 ± 41	0.65 ± 0.05
15.9	99 ± 2	92 ± 3	−32.1 ± 1.7	458 ± 36	0.65 ± 0.05
**PLGA+PEG**					
0.0	---	80 ± 3	−30 ± 2	446 ± 40	0.48 ± 0.04
5.0	99 ± 3	82 ± 2	−26.8 ± 1.3	435 ± 29	0.69 ± 0.06
10.0	99 ± 3	82 ± 1	−33.0 ± 1.6	493 ± 38	0.66 ± 0.06
15.0	98 ± 2	80 ± 2	−30.2 ± 1.6	466 ± 45	0.68 ± 0.07

**Table 2:** Encapsulation efficiency (% EE), synthesis yield (% R), zeta potential (ζ), hydrodynamic diameter (d_H_), and polydispersity index (PDI) values obtained in the preparation of blank and 7,8-DHF-loaded polymeric NPs, for different initial amounts of flavone (mg) used in the synthesis. The experimental values are expressed as the mean ± SD (*n* = 3).

PLGA					
mg 7,8-DHF	% EE	% R	ζ (mV)	d_H_ (nm)	PDI
0	---	90 ± 3	−32.5 ± 1.4	442 ± 10	0.433 ± 0.017
2.5	91 ± 2	93 ± 2	−28.1 ± 1.0	193 ± 9 520 ± 30	0.50 ± 0.06
5.0	83 ± 2	92 ± 3	−31.2 ± 0.8	187 ± 12 592 ± 25	0.40 ± 0.04
10.0	88 ± 3	91 ± 3	−35.6 ± 0.9	160 ± 13 560 ± 30	0.62 ± 0.05
15.0	91 ± 3	93 ± 3	−29.6 ± 1.4	165 ± 3 549 ± 7	0.52 ± 0.08
**PLGA-PEG**					
0.0	---	88 ± 2	−28.7 ± 1.8	424 ± 30	0.51 ± 0.04
5.0	92 ± 2	90 ± 3	−30.2 ± 1.5	450 ± 35	0.62 ± 0.06
10.0	92 ± 3	88 ± 2	−29.1 ± 1.2	463 ± 35	0.69 ± 0.08
15.0	89 ± 2	91 ± 2	−31.9 ± 1.8	478 ± 41	0.64 ± 0.05
**PLGA+PEG**					
0.0	---	80 ± 1	−30 ± 2	436 ± 29	0.48 ± 0.04
5.0	97 ± 3	82 ± 1	−29.3 ± 1.4	415 ± 33	0.45 ± 0.03
7.5	90 ± 2	85 ± 2	−34.0 ± 1.3	453 ± 32	0.51 ± 0.07
10.0	91 ± 2	80 ± 2	−34.1 ± 1.8	489 ± 42	0.59 ± 0.08

**Table 3:** Encapsulation efficiency (% EE), synthesis yield (% R), zeta potential (ζ), hydrodynamic diameter (d_H_), and polydispersity index (PDI) values obtained in the preparation of blank and BA-loaded polymeric NPs at different initial amounts of flavone (mg) used in the synthesis. The experimental values are expressed as the mean ± SD (*n* = 3).

PLGA					
mg BA	% EE	% R	ζ (mV)	d_H_ (nm)	PDI
0	---	90 ± 3	−32.5 ± 1.4	442 ± 10	0.433 ± 0.017
2.5	98 ± 3	87 ± 2	−30.1 ± 1.5	431 ± 22	0.51 ± 0.04
5.1	97 ± 3	94 ± 3	−28 ± 1.1	455 ± 39	0.69 ± 0.05
9.9	98 ± 2	88 ± 2	−32 ± 1.4	428 ± 30	0.58 ± 0.04
15.6	96 ± 3	89 ± 3	−35 ± 1.4	482 ± 32	0.65 ± 0.08
**PLGA-PEG**					
0.0	---	88 ± 2	−28.7 ± 1.8	424 ± 30	0.51 ± 0.04
2.5	98 ± 3	90 ± 3	−30.0 ± 1.6	441 ± 29	0.47 ± 0.06
5.4	99 ± 2	90 ± 2	−29.7 ± 1.2	399 ± 36	0.48 ± 0.06
10.2	99 ± 2	88 ± 2	−28.9 ± 1.5	445 ± 32	0.50 ± 0.04
16.0	99 ± 3	87 ± 3	−31.6 ± 1.3	487 ± 41	0.69 ± 0.07
**PLGA+PEG**					
0.0	---	80 ± 1	−30 ± 2	436 ± 29	0.48 ± 0.04
5.2	96 ± 2	85 ± 1	−27.3 ± 1.5	399 ± 42	0.54 ± 0.06
10.0	98 ± 3	83 ± 2	−31.6 ± 1.7	459 ± 34	0.57 ± 0.08
15.5	94 ± 2	85 ± 2	−35.1 ± 1.3	431 ± 37	0.70 ± 0.08

**Table 4:** Encapsulation efficiency (% EE), synthesis yield (% R), zeta potential (ζ), hydrodynamic diameter (d_H_), and polydispersity index (PDI) values obtained in the preparation of blank and LU-loaded polymeric NPs at different initial amounts of flavone (mg) used in the synthesis. The experimental values are expressed as the mean ± SD (*n* = 3).

PLGA					
mg LU	% EE	% R	ζ (mV)	d_H_ (nm)	PDI
0	---	80 ± 3	−32.5 ± 1.4	442 ± 10	0.433 ± 0.017
2.5	98 ± 3	75 ± 2	−30.7 ± 2.5	315 ± 2.6	0.37 ± 0.03
5.0	98 ± 2	78 ± 3	−28.8 ± 0.6	397 ± 19	0.38 ± 0.01
10	98 ± 2	74 ± 2	−25.6 ± 0.8	520 ± 95	0.56 ± 0.02
15	99 ± 2	82 ± 2	−30.7 ± 2.4	492 ± 55	0.64 ± 0.08
**PLGA-PEG**					
0.0	---	81 ± 2	−28.7 ± 1.8	424 ± 3 0	0.51 ± 0.04
2.5	98 ± 3	79 ± 2	−34.6 ± 0.6	454 ± 43	0.49 ± 0.05
5.0	99 ± 2	77 ± 3	−35.4 ± 0.5	443 ± 33	0.69 ± 0.05
10	99 ± 2	80 ± 3	−29.4 ± 0.7	459 ± 30	0.60 ± 0.03
15	99 ± 3	82 ± 2	−31.5 ± 0.4	420 ± 31	0.76 ± 0.04
**PLGA+PEG**					
0.0	---	78 ± 2	−30 ± 2	436 ± 29	0.48 ± 0.04
2.5	97 ± 3	82 ± 2	−30.3 ± 0.3	406 ± 32	0.49 ± 0.04
5.0	99 ± 3	80 ± 3	−29.2 ± 0.2	469 ± 20	0.41 ± 0.02
10	99 ± 3	78 ± 3	−31.2 ± 0.2	460 ± 42	0.41 ± 0.04
15	99 ± 2	80 ± 2	−32.9 ± 0.4	427 ± 24	0.64 ± 0.04

**Table 5:** EC_50_ values for the free ascorbic acid and the free flavones used in this work, as well as those values corresponding to the flavone-loaded NPs.

Antioxidant	EC_50_ (µM)
Free ascorbic acid	15.9 ± 1.1
Free 7-HF	>2000
PLGA loaded 7-HF	No activity
PLGA-PEG loaded 7-HF	No activity
PLGA+PEG loaded 7-HF	No activity
Free 7,8-DHF	13.8 ± 1.8
PLGA loaded 7,8-DHF	20.8 ± 2.5
PLGA-PEG loaded 7,8-DHF	22.5 ± 1.6
PLGA+PEG loaded 7,8-DHF	17.1 ± 1.1
Free baicalein	7.9 ± 1.4
PLGA loaded baicalein	13.0 ± 1.1
PLGA-PEG loaded baicalein	14.6 ± 1.6
PLGA+PEG loaded baicalein	11.9 ± 0.8
Free luteolin	5.8 ± 1.1
PLGA loaded luteolin	5.7 ± 0.8
PLGA-PEG loaded luteolin	4.1 ± 1.2
PLGA+PEG loaded luteolin	5.1 ± 1.0

**Table 6:** Minimum inhibitory concentrations (MIC, µg/mL) of polyphenolic compounds formulated as free or as loaded nanoparticles, with blank nanoparticles used as controls.

Microorganisms					
		Gram-Positive		Gram-Negative	
Compound	Formulation	*Bacillus* *subtilis* CCM 2216	*Staphylococcus aureus* ATCC 27697	*Escherichia coli* CECT 101	*Pseudomonas aeruginosa* ATCC 27853
**Luteolin**	Free (µg mL^−1^)	32–64	4–8	>512	>512
	Blank PLGA NPs (mg mL^−1^)	>1.32	>1.32	>1.32	>1.32
	Loaded PLGA NPs (µg mL^−1^) *	32 (0.083)	4–8 (0.010–0.021)	128–512 (0.33–1.32)	>512 (>1.32)
	Blank PLGA+PEG NPs (mg mL^−1^)	>3.54	0.22–0.44	>3.54	>3.54
	Loaded PLGA+PEG NPs (µg mL^−1^) *	>512 (>3.54)	4–8 (0.028–0.055)	64 (0.44)	>512 (>3.54)
	Blank PLGA-PEG NPs (mg mL^−1^)	>2.38	>2.38	>2.38	>2.38
	Loaded PLGA-PEG NPs (µg mL^−1^) *	256 (1.19)	32–64 (0.15–0.30)	>512 (>2.38)	>512 (>2.38)
**Baicalein**	Free (µg mL^−1^)	16	4–8	8–16	8
	Blank PLGA NPs (mg mL^−1^)	>1.42	>1.42	>1.42	>1.42
	Loaded PLGA NPs (µg mL^−1^) *	32–64 (0.089–0.18)	16–32 (0.044–0.089)	64 (0.18)	32 (0.089)
	Blank PLGA+PEG NPs (mg mL^−1^)	>1.84	0.23–0.46	>1.84	>1.84
	Loaded PLGA+PEG NPs (µg mL^−1^) *	32–64 (0.12–0.23)	16–32 (0.058–0.12)	64–128 (0.23–0.46)	32 (0.12)
	Blank PLGA-PEG NPs (mg mL^−1^)	>1.25	>1.25	>1.25	>1.25
	Loaded PLGA-PEG NPs (µg mL^−1^) *	32–64 (0.078–0.16)	64 (0.16)	64 (0.16)	32 (0.078)
**7-HF**	Free (µg mL^−1^)	>512	>512	>512	>512
	Blank PLGA NPs (mg mL^−1^)	>1.56	>1.56	>1.56	>1.56
	Loaded PLGA NPs (µg mL^−1^) *	>512 (>1.56)	>512 (>1.56)	>512 (>1.56)	>512 (>1.56)
**7,8-DHF**	Free (µg mL^−1^)	>512	8–16	>512	>512
	Blank PLGA NPs (mg mL^−1^)	>3.02	>3.02	>3.02	>3.02
	Loaded PLGA NPs (µg mL^−1^) *	>512 (>3.02)	8–16 (0.047–0.094)	>512 (>3.02)	>512 (>3.02)

* Displayed in parentheses is the concentration of NPs (mg mL^−1^) in the microplate well to achieve the indicated drug concentration.

## Data Availability

The original contributions presented in this study are included in the article/Supplementary Materials. Further inquiries can be directed to the corresponding authors.

## Supplementary Materials

The following supporting information can be downloaded at: https://www.mdpi.com/article/10.3390/pharmaceutics18060676/s1, Figure S1: Representative DLS histograms. (a) Blank PLGA NPs; (b) 7-HF-loaded PLGA-PEG (5 mg); (c) BA-loaded PLGA+PEG NPs (10 mg); (d) LU-loaded PLGA NPs (5 mg); Figure S2: FT-IR spectra of free flavones 7-HF, 7,8-DHF, BA, and LU; blanks (unloaded NPs of PLGA, PLGA-PEG and PLGA+PEG); and some NPs loaded with flavones (PLGA 7-HF, PLGA 7,8-DHF, PLGA BA, PLGA LU, PLGA-PEG 7-HF, PLGA-PEG BA, PLGA+PEG 7-HF, PLGA+PEG BA); Table S1: Main peaks and assignments observed in the FTIR spectra; Figure S3: Release rate constant values for 7-HF and BA, at different pH values, in the different types of polymeric NPs. Each experiment was repeated thrice independently. Error bars represent standard deviation values. Tests were done using the GraphPad Prism 8.0.1 software program (San Diego, CA, USA). Statistics were determined with a two-way ANOVA and Tukey’s multiple comparisons test. * *p* < 0.05, ** *p* < 0.01, *** *p* < 0.001, and **** *p* < 0.0001; Figure S4: Release rate constant values for 7-HF and BA, at different pH values in the different types of polymeric NPs. Each experiment was repeated thrice independently. Error bars represent standard deviation values. Tests were done using the GraphPad Prism 8.0.1 software program (San Diego, CA, USA). Statistics were determined with a two-way ANOVA and Sidak’s multiple comparisons test. * *p* < 0.05, ** *p* < 0.01, *** *p* < 0.001, and **** *p* < 0.0001; Figure S5: Release rate constant values for 7-HF, 7,8-DHF, BA, and LU at different pH values, in the PLGA polymeric NPs. Each experiment was repeated thrice independently. Error bars represent standard deviation values. Tests were done using the GraphPad Prism 8.0.1 software program (San Diego, CA, USA). Statistics were determined with a two-way ANOVA and Sidak’s multiple comparisons test. * *p* < 0.05, ** *p* < 0.01, *** *p* < 0.001, and **** *p* < 0.0001.

## Abbreviations

The following abbreviations are used in this manuscript:

NPs	Nanoparticles
PLGA	Poly(lactic-co-glycolic) acid
PEG	Polyethylene glycol
PLGA-PEG	(Polyethylene glycol)methyl ether-block-Poly(lactide-co-glycolide)
PVA	Polyvinyl alcohol
BA	Baicalein
7-HF	7-hydroxyflavone
LU	Luteolin
7,8-DHF	7,8-dihydroxyflavone
CECT	Spanish Culture Collection of Type Cultures
ATCC	American Type Culture Collection, Manassas
CCM	Czech Collection of Microorganisms
Ζ	Zeta potential
TEM	Transmission electron microscopy
EE	Encapsulation efficiency
DPPH·	2,2-diphenyl-1-picrylhydrazyl
LC	Loaded capacity
CLSI	Clinical and Laboratory Standards Institute
MIC	Minimal inhibitory concentration
DMSO	Dimethyl sulfoxide
CAMHB	Mueller Hinton II broth
TSA	Tryptic soy agar
d_H_	Hydrodynamic diameter
PDI	Polydispersity index
