# Supplementary material for: Plant-Based Flavones of Therapeutic Interest Loaded into Polymeric Nanoparticles
Source: Pharmaceutics. 2026 May 29;18(6):676. doi: 10.3390/pharmaceutics18060676 (PMC13304834; doi:10.3390/pharmaceutics18060676)
Supplement: Supplementary file 1 [file pharmaceutics-18-00676-s001.zip › pharmaceutics-4283847-supplementary.pdf]

SUPPLEMENTARY MATERIALS

Plant-based flavones of therapeutic interest loaded into polymeric nanoparticles

Cecilia Espíndola [1], Ira Wirth [1], Victoria Isabel Martín [1], Eva Bernal [1], José Antonio Lebrón [1], María Luisa Moyá [1], Rafael R. de la Haba [2], Cristina Sánchez-Porro [2], Antonio Ventosa [2], Carmen M. Granados-Carrera [3], Sara Molina [1], Alvaro Hidalgo [1], Manuel López-López [4,*], Pilar López-Cornejo [1,*], and Francisco José Ostos [1]

[1] Department of Physical Chemistry, University of Seville, C/ Profesor García González 1, 41012 Seville, Spain; caced0617@gmail.com (C.E.); ira.wirth@uni-hamburg.de (I.W.); vmartin1@us.es (V.I.M.); evabernal@us.es (E.B.); jlebron@us.es (J.A.L.); moya@us.es (M.L.M.); saramolinavg@gmail.com (S.M.); alvhidyer@alum.us.es (A.H.); pcornejo@us.es (P.L.-C.); fostos@us.es (F.J.O.)

[2] Department of Microbiology and Parasitology, University of Seville, C/Profesor García González 2, 41012 Seville, Spain; rrh@us.es (R.R.H.); sanpor@us.es (C.S.-P.); ventosa@us.es (A.V.)

[3] Department of Chemical Engineering, Faculty of Chemistry, University of Seville, C/Profesor García González 1, 41012 Seville, Spain; cargracar@alum.us.es

[4] Department of Chemical Engineering, Physical Chemistry and Materials Science, Faculty of Experimental Sciences, Campus de El Carmen, Avda. de las Fuerzas Armadas s/n, 21007 Huelva. Spain; manuel.lopez@diq.uhu.es (M.L.-L.)

* Correspondence: manuel.lopez@diq.uhu.es (M.L.-L.); pcornejo@us.es (P.L.-C.); Tel.: +34-955-421-005 (P.L.-C.)

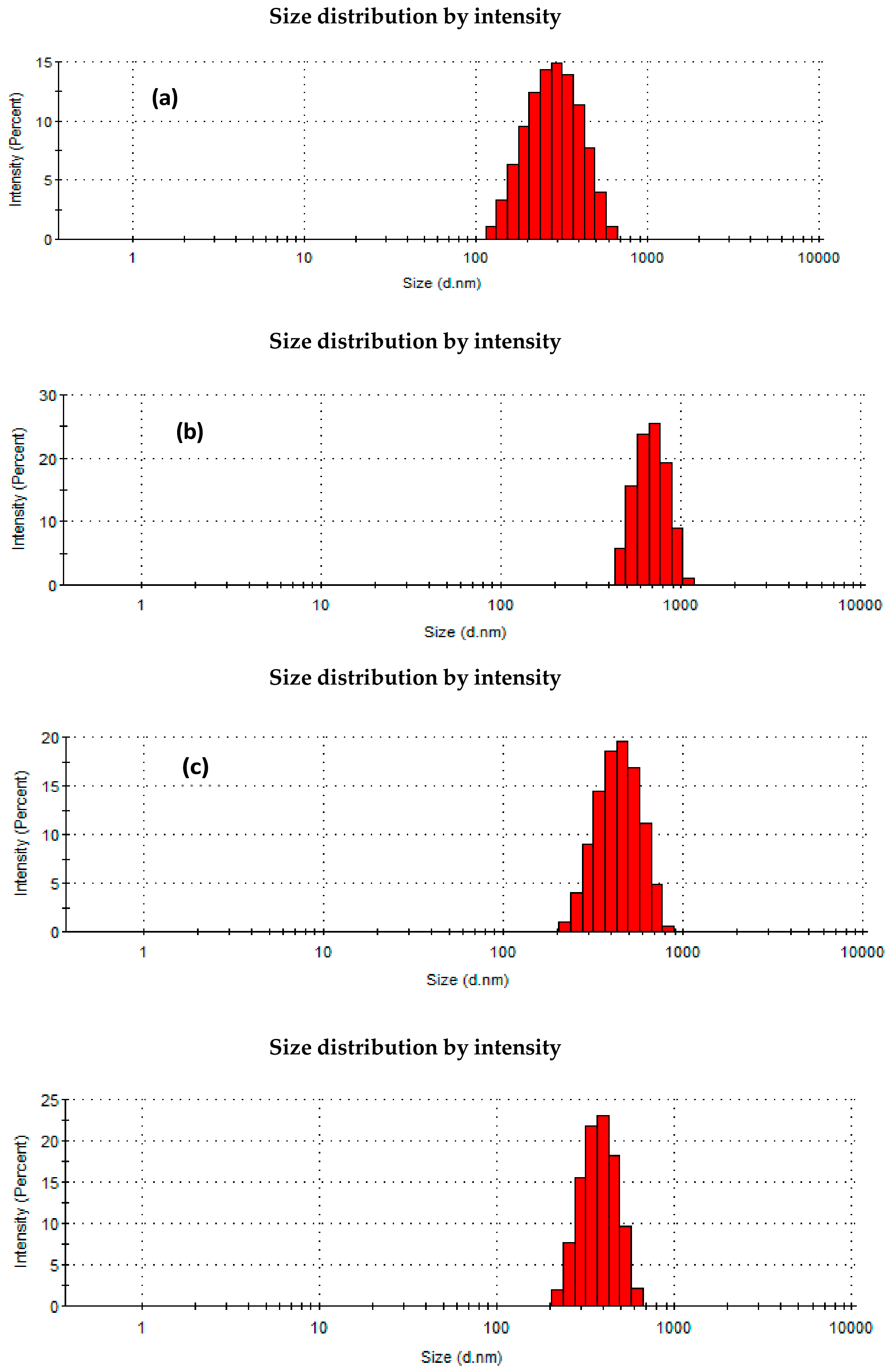

Figure S1. Representative DLS histograms. (**a**) Blank PLGA NPs; (**b**)7-HF-loaded PLGA-PEG (5 mg); (**c**) BA-loaded PLGA+PEG NPs (10 mg); (**d**) LU-loaded PLGA NPs 5 mg).

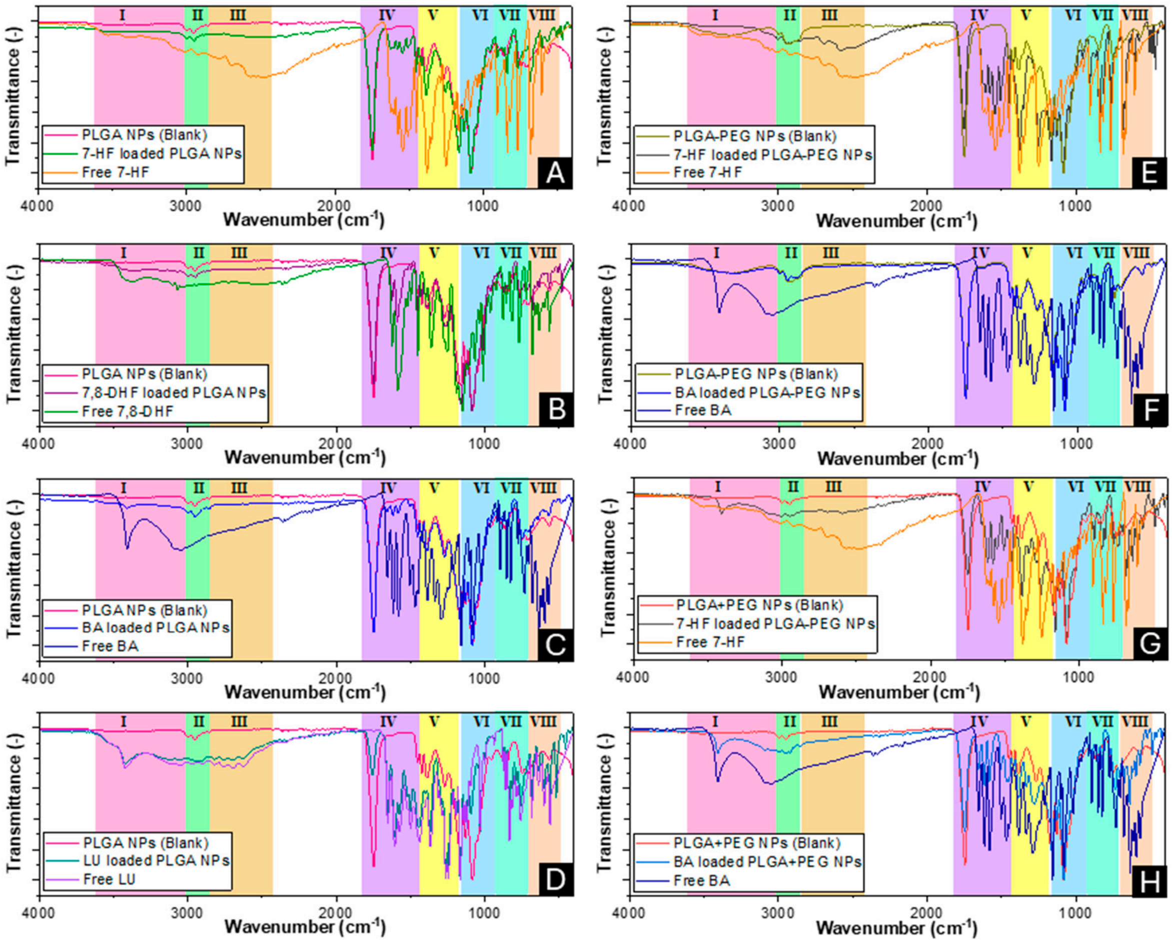

Figure S2. FTIR spectra of free flavones 7-HF, 7,8-DHF, BA and LU; Blanks: unloaded NPs of PLGA, PLGA-PEG and PLGA+PEG; and some NPs loaded with the flavones (PLGA 7-HF, PLGA 7,8-DHF, PLGA BA, PLGA LU, PLGA-PEG 7-HF, PLGA-PEG BA, PLGA+PEG 7-HF, PLGA+PEG BA). Figures A–H show the different systems studied, comparing the spectra of the loaded nanoparticles with those of the free drug and those of the unloaded nanoparticles (Blank).

Table S1. Main peaks and assignments observed in the FTIR spectra

System (CH-LG)		Peak (cm^{-1})	Assignation	References
Signal	I	3600 – 3050	O-H stretching from hydroxyl groups<br>Intermolecular hydrogen bonding	[1-6]
	II	3075 – 2750	Aliphatic C-H stretching of CH_3/CH_2	
	III	2750 – 2400	Weak overtone/combination bands	
	IV	1800 – 1400	Strong ester carbonyl (C=O) stretching<br>Aromatic C=C vibrations	
	V	1400 – 1150	CH_3/CH_2 deformation<br>C-O and C-O-C ester bond stretching	
	VI	1150 – 900	C-O-C ester bond vibrations<br>Trans vibrations of conjugated double bonds<br>Alcohol C-O bond stretching	
	VII	900 – 700	-CH rocking<br>Aromatic ring deformation<br>Skeletal vibration	
	VIII	700 – 500	Skeletal vibration<br>Aromatic ring bending	

References:

1. Hajian, M.; Erfani-Moghadam, V.; Arabi, M.S.; Soltani, A.; Shahbazi, M. A Comparison between Optimized PLGA and CS-Alg-PLGA Microspheres for Long-Lasting Release of Glatiramer Acetate. *J. Drug Deliv. Sci. Technol.* **2023**, *82*, 104355, doi:10.1016/j.jddst.2023.104355.

2. Zafar, A.; Alruwaili, N.K.; Imam, S.S.; Alsaidan, O.A.; Alharbi, K.S.; Alzarea, S.I.; Yasir, M.; Afzal, M.; Alshehri, S.; Alanazi, A.S. Bioactive Luteolin Entrapped Chitosan-PLGA Nanoparticles: Formulation Optimization to In-Vivo Preclinical Evaluation. *J. Clust. Sci.* **2023**, *34*, 437–449, doi:10.1007/s10876-022-02232-7.

3. Zhang, M.; Huang, Z.; Wang, X.; Liu, X.; He, W.; Li, Y.; Wu, D.; Wu, S. Personalized PLGA/BCL Scaffold with Hierarchical Porous Structure Resembling Periosteum-Bone Complex Enables Efficient Repair of Bone Defect. *Advanced Science* **2024**, *11*, doi:10.1002/advs.202401589.

4. Manaka, A.; Uvarani, R. Molecular Structure, Spectroscopic, Electronic and Physicochemical Properties of 7,8-Dihydroxyflavone Hydrate Compound Using First Theory Principle. *Journal of the Indian Chemical Society* **2023**, *100*, 101034, doi:10.1016/j.jics.2023.101034.

5. Kitmür, İ.; Mesci, S.; Ay, E.B.; Yıldırım, T.; Çiftçi, G.Y. The First 6-Hydroxyflavone and 7-Hydroxyflavone Decorated Monospiro Cyclotriphosphazene Compounds: Synthesis, Characterization and Biological Activity Studies. *Inorganica Chim. Acta* **2025**, *587*, 122812, doi:10.1016/j.ica.2025.122812.

6. Sharma, D.K.; Pattnaik, G.; Behera, A. Preparation and In-Vitro, in-Vivo Characterisation of Pioglitazone Loaded Chitosan/PEG Blended PLGA Biocompatible Nanoparticles. *J. Biomater. Sci. Polym. Ed.* **2022**, *33*, 1623–1643, doi:10.1080/09205063.2022.2068947.

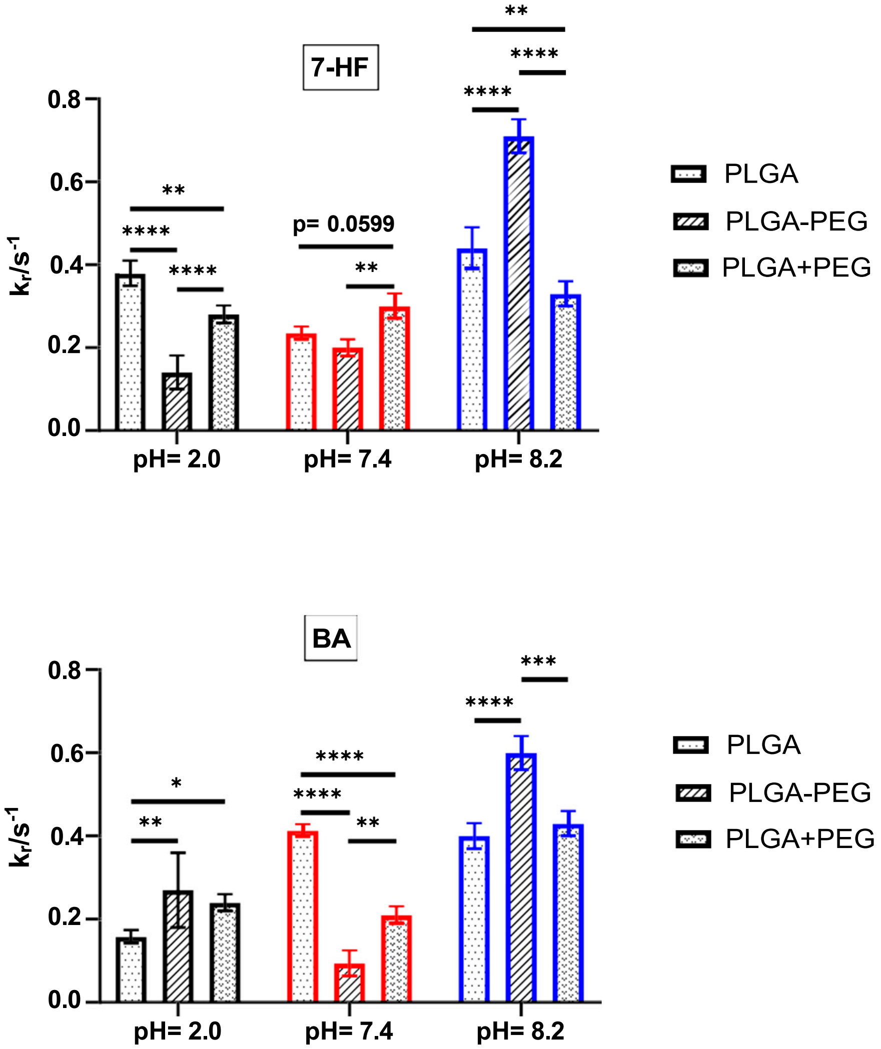

Figure S3. Release rate constant values for 7-HF and BA, at different pHs, in the different types of polymeric NPs. Error bars represent standard deviation values. Tests were done using the GraphPad Prism 8.0.1 software program (San Diego, CA). Statistics were determined with a two-way ANOVA Tukey´s multiple comparisons test. *P < 0.05, **P < 0.01, ***P < 0.001 and ****P < 0.0001.

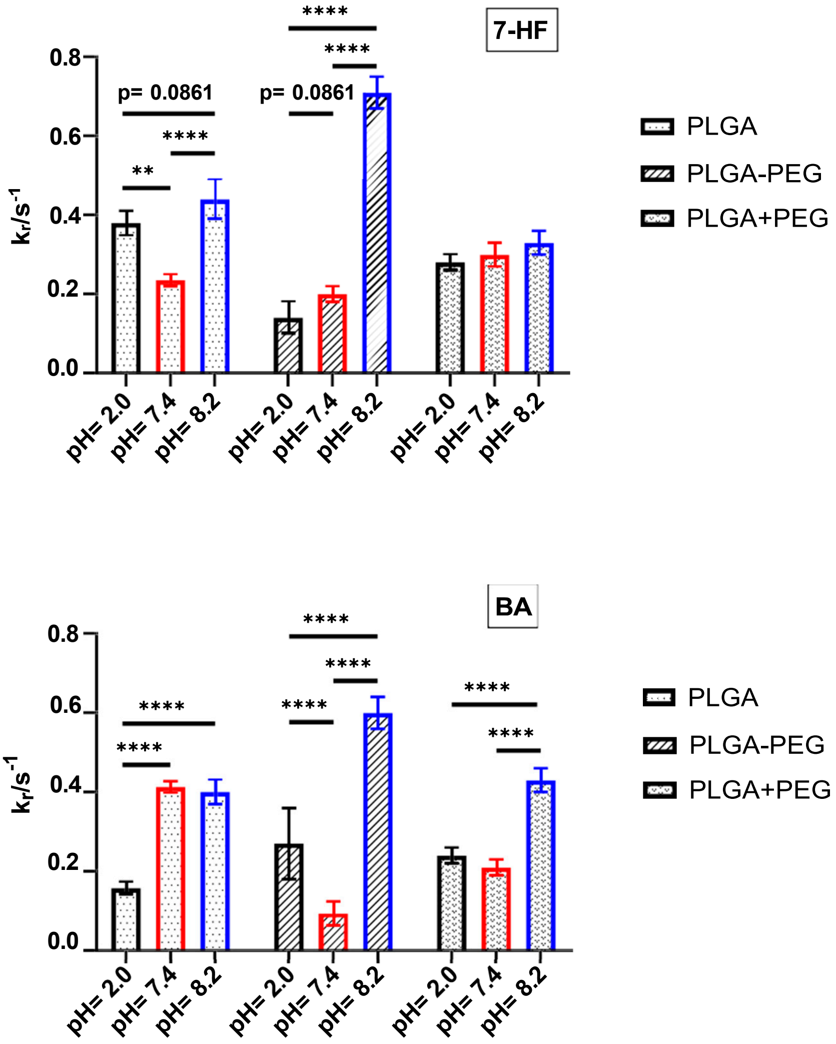

Figure S4. Release rate constant values for 7-HF and BA, at different pHs, in the different types of polymeric NPs. Error bars represent standard deviation values. Tests were done using the GraphPad Prism 8.0.1 software program (San Diego, CA). Statistics were determined with a two-way ANOVA Sidak´s multiple comparisons test. *P < 0.05, **P < 0.01, ***P < 0.001 and ****P < 0.0001.

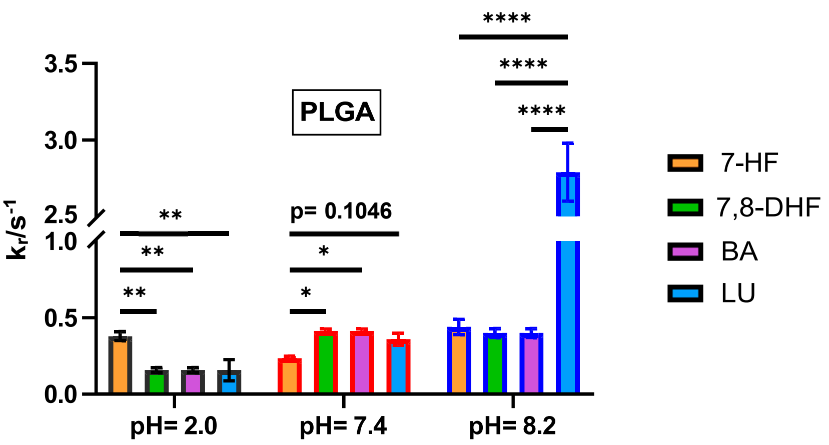

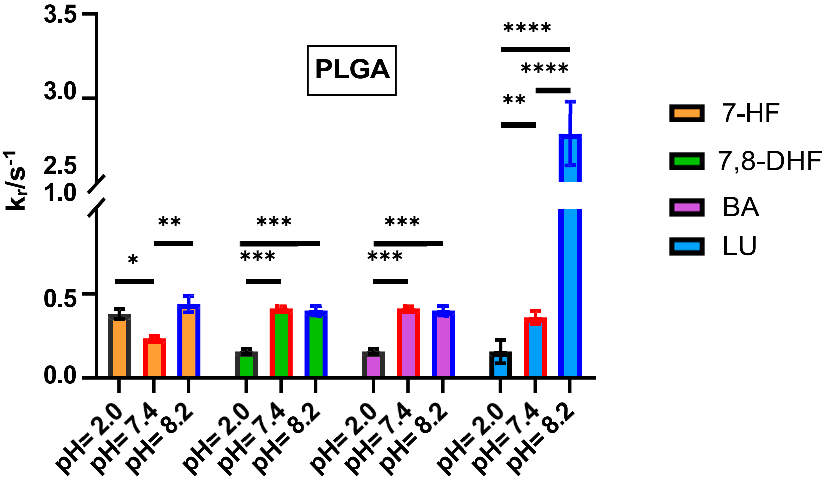

Figure S5. Release rate constant values for 7-HF, 7,8-DHF, BA, and LU at different pHs, in the PLGA polymeric NPs. Error bars represent standard deviation values. Tests were done using the GraphPad Prism 8.0.1 software program (San Diego, CA). Statistics were determined with a two-way ANOVA Sidak´s multiple comparisons test. *P < 0.05, **P < 0.01, ***P < 0.001 and ****P < 0.0001.
